# Perceptions of Patients and Their Families Regarding Limitation of Therapeutic Effort in the Intensive Care Unit

**DOI:** 10.3390/jcm10214900

**Published:** 2021-10-24

**Authors:** Juan Carlos Muñoz Camargo, Antonio Hernández-Martínez, Julián Rodríguez-Almagro, María Laura Parra-Fernández, María del Carmen Prado-Laguna, Mairena Martín

**Affiliations:** 1 Department of Nursing, Physiotherapy and Occupational Therapy, Faculty of Nursing of Ciudad Real, Universidad de Castilla-La Mancha, 13071 Ciudad Real, Spain; Juancarlos.munoz@uclm.es (J.C.M.C.); Antonio.hmartinez@uclm.es (A.H.-M.); julianj.rodriguez@uclm.es (J.R.-A.); marialaura.parra@uclm.es (M.L.P.-F.); carmina.prado@uclm.es (M.d.C.P.-L.); 2 Department of Inorganic, Organic Chemistry and Biochemistry, Faculty of Nursing of Ciudad Real, Regional Center of Biomedical Research (CRIB), Universidad de Castilla-La Mancha, 13091 Ciudad Real, Spain

**Keywords:** appropriateness of therapeutic effort, death, quality of life, end-of-life care

## Abstract

Objective: Our objective was to determine and describe the opinion and attitudes of patients and their families regarding the limitation of therapeutic effort and advanced directives in critical patients and whether end-of-life planning occurs. Religious affiliation, education level, and pre-admission quality of life were also evaluated to determine whether they may influence decisions regarding appropriate therapeutic effort. Methods: A prospective, observational and descriptive study, approved by the center’s ethical committee, was carried out with 257 participants (94 patients and 163 family members) in the intensive care unit (ICU). A questionnaire regarding the opinions of patients and relatives about situations of therapeutic appropriateness in case of poor prognosis or poor quality of life was used. The questionnaire had three sections. In the first section, sociodemographic features were investigated. In the second section, information was collected on the quality of life and functional situation before ICU admission (taking as a reference the situation one month before admission) assessed by the Karnofsky scale, Barthel index, and the PAEEC scale (Project for the Epidemiological Analysis of Critical Care Patients). The third section aimed to determine whether the family knew the patient’s opinion regarding his/her end of life. Results: Of those interviewed, 62.2% would agree to limit treatment in case of poor prognosis or poor quality of future life. In contrast, 37.7% considered that they should fight for life, even if it is irretrievable. Only 1.6% had advanced directives registered, 43.9% of the participants admitted deterioration in their quality of life before ICU admission, 18.2% with moderate-severe deterioration. Our study shows that the higher the educational level, the lower the desire to fight for life when it is irretrievable and the greater the agreement to limit treatment. Besides, those participants not affiliated with a religion were significantly less likely to fight for life, including when irretrievable, than Catholics and were more likely to agree to limit treatment. Conclusions: More than half of the participants would agree to limit treatment in the case of a poor prognosis. Our results indicate that patients do not prepare for the dying process well in advance. Religion and educational level were determining factors for the choice of procedures at the end of life, both for patients and their families.

## 1. Introduction

Death is a frequent event in the intensive care unit (ICU) because of the nature of the lesions, diseases, and the sometimes unpredictable clinical responses of critical patients. The process of dying is formalized once it becomes clear that the patient will not recover and when life support measures are no longer beneficial for their recovery [1]. In ICUs worldwide, a limitation of therapeutic effort (LTE) protocol is implemented. LTE consists in not applying pharmacological therapies or instrumental procedures, more or less invasive, considered (by the scientific community) adequate to treat diseases that put life at risk [2]. However, it is difficult to determine the incidence of this practice due to the variability in the definition of the concept, the measures that are considered susceptible to its practice, and the different cultural, ethical, and legal contexts of the countries in which it is applied. In Europe, the “Ethicus Study” found that 9.8% of patients admitted to the ICU died within the context of LTE [3].

Treatments in the ICU are increasingly more sophisticated to maintain the lives of critical patients [4]; however, in many cases, death is associated with the withdrawal of treatments after technological interventions and systems have been exhausted and body systems are not responding effectively to treatment [5]. Although technology has contributed to modifying the conditions in which the death of these patients occurs and delayed it, many of the interventions that tend to keep the patient alive have been used unreasonably [6].

LTE avoids falling into therapeutic obstinacy [7,8]. The principals of autonomy, non-maleficence, beneficence and justice are the ethical principles on which all-care relationships, including LTE, must be based today [9,10]. In agreement, basic treatment and care measures are adapted to the patient’s condition [11]. Given that quality of life has been an important predictor of survival, it should be considered part of the routine evaluation of clinical processes [12].

To verify the efficacy of modern treatments, not only are results related to life expectancy and survival rate being considered but aspects that are related to the subjective evaluations of the patient regarding their well-being are also increasingly being included [13]. In the last century, a model focused on the patient’s right to autonomy and receiving adequate information has been implemented which has led to empowerment in clinical decision making [14].

Individual planning of end-of-life care, including open communication about a person’s preferences and attitudes, can facilitate dignity and quality of life for patients and their families [15]. However, in the context of end-of-life care, decisions are often uncertain, and in some cases, they may fail to consider the patient’s own values and wishes fully [16].

It has been shown that both the general population and healthcare professionals do not have sufficient knowledge on this subject [17]. To achieve participation in health and end-of-life decisions, not only the knowledge of professionals is required, but also a legal and terminological consensus in which the opinions of individuals are included [18].

In Spain, the legal framework for LTE is contained in several laws: The Council of Europe Convention for the protection of human rights [19], Law 41/2002, basic regulation of patient autonomy [20], Royal Decree 124/2007 which regulates the National Registry of advance directives [21], and in the Castilla-La Mancha autonomous community, where the present study was done, Decree 15/2006 regarding the Registry of Advance Wills [22]. These legal aspects differ from country to country. The most important criteria in Spain to establish LTE are evaluating the case and determining the patient’s preferences. Therefore, if the patient’s preferences cannot be expressed, then the health professional should determine if they have made advance directives. If not, the medical team may act for the patient’s benefit, agreeing on the measures with direct family members. Unfortunately, decisions made by family members do not often correspond to the patient’s choice, since they do not know it [7,8].

Since ancient times, the health-disease process has become the object of religious beliefs and practices. From different religious points of view, the use of medical techniques to prolong life in patients who inevitably have to die, i.e., therapeutic obstinacy, is generally discouraged, and there is general agreement that excessive use of technology does not form part of good practice [23]. Some studies that address ethical decisions in ICU show two different facets of influence: an English perspective, where the rights and autonomy of the patient, informed consent, freedom, and principles of justice predominate; an Eastern one, where other beliefs based on traditions predominate, emphasizing the importance of the family group beyond the patient’s autonomy, as is also seen in Europe [24].

It is necessary to investigate how citizens experience the possibility of death, their opinion about organ donation, advanced directives, limitation of therapeutic effort, refusal of certain treatments, and place where they want to be treated at the end of life [25,26,27], in short, how they want to be treated in those final moments, what values they want to be respected, how they want healthcare professionals, their families, and representatives to be involved in clinical decision making, when they are no longer capable of making treatment decisions [28].

Therefore, the present study aimed (i) to describe the opinion and attitude of ICU patients and family members regarding LTE and advanced directives in the critical patient; (ii) to assess whether planning for the end-of-life process exists; and (iii) to evaluate the influence, if any, of religious affiliation, educational level, and quality of life before ICU admission on the opinions regarding LTE.

## 2. Methods

### 2.1. Type of Study and Setting

A prospective descriptive unicentric observational study was conducted. The setting was in the intensive care unit (ICU) of the Hospital General Universitario of Ciudad Real (Spain).

### 2.2. Study Population and Sample Size

The study population was patients and family members admitted to the hospital ICU during 2019. A non-probabilistic and consecutive sampling of patients admitted to the ICU and agreed to participate in the study was carried out. To calculate the sample size, the GRANMO sample size calculator was used. A sample of 257 participants was estimated for a confidence level of 95% and a precision of ± 5 percentage units, for a population percentage subject to the limitation of life support treatment (LLST) of around 30%. The percentage of dropouts or non-response was estimated as 20%.

### 2.3. Inclusion and Exclusion Criteria

Patients 18 years and over, and their family members, admitted to the ICU for more than 24 h were included in the study cohort.

Patients with acute coronary syndrome and those admitted to the ICU to monitor treatments or cannulation of the venous lines were excluded. These exclusion criteria were established because these patients had low hospital mortality in the medium term as compared to the rest of critical patients.

### 2.4. Measurement Instruments and Variables

A questionnaire specifically designed for this research was used in which the views of relatives and patients were assessed regarding situations of treatment limitation in the event of a poor prognosis or poor quality of life. The survey consisted of three sections. In the first section, sociodemographic variables were collected: age, sex, religion, educational level, the reason for ICU admission, as well as previous admissions to the ICU. In the second section, information was collected on the quality of life and functional situation prior to ICU admission (using the month prior to admission as reference) as assessed by the Karnofsky scale [29], the Barthel index [30], and the PAEEC (Project for the Epidemiological Analysis of Critical Care Patients) scale [31]. The Karnofsky scale, an independent predictor of mortality in oncological but also in non-oncological diseases, is used to determine a patient’s capacity in terms of daily life activities. This scale is used for clinical decision making and to assess the impact of treatment and disease progression. The score ranges from 100 (normal with no complaints or signs of illness) to zero (deceased). A Karnofsky less than or equal to 50 indicates a high risk of death in the following 6 months. The Barthel Index is a widely used instrument to assess functional status, assigning each patient a score based on the degree of dependence on it to perform a series of basic activities. The group “Project for the Epidemiological Analysis of Critical Care Patients” (PAEEC based on its Spanish Name) developed a health-related quality of life questionnaire, based on a database from a multicenter study in which 87 intensive medicine services participated. This questionnaire has been validated and analyzes three dimensions: basic physiological activities; activities of daily living, including physical, work, and social activity; and emotional status. It comprises 15 items with their corresponding score, and the global score can range from 0 (normal quality of life or without limitations) to 29 points (maximum deterioration in the quality of life). The third section aims to determine if the family know the patient’s opinion regarding: (a) wishes to limit their treatment in case of poor prognosis or poor quality of life; (b) if they had expressed their willingness to donate their organs, both for and against and, if so, if they had made it official; (c) if they had appointed a vital representative, that is, a person who would decide for him/her in situations in which he/she was not capable; (d) if they had a living will or advance directives, (e) if they had a legal will, and (f) if they should be considered in the decision-making process for procedures and treatments.

### 2.5. Procedures

Data on the opinions of patients or their families were collected through an interview. The first choice was to interview the patient. If the patient was unable to participate or answer interview questions, the interview was carried out with the direct family of the patient.

The interview was carried out between the second and seventh day of ICU admission to give patients and their families time to adapt to the patient’s situation, their treatment with the healthcare personnel, and the operating regulations of the unit.

The interview was conducted by the same interviewer, a nurse with more than 25 years of experience in the ICU to avoid misinterpretation both in the questions and in the answers. Moreover, quality of life, dependency, and clinical situation measurements were carried out with validated instruments with which the interviewer was familiar since these indices are used daily in the ICU. The interviewer did not decide whether the patient is capable or not to respond to the questionnaire. First, the interviewer tried to directly interview the patient and only when the patient was under sedation or coma, the interview was performed with the corresponding family member. In addition, apart from requesting informed consent, it was made clear that the interview was not related to any aspects that affected the prognosis, diagnosis, or treatment of the illness. The interview was conducted in a comfortable place, where family members could sit. To avoid excessive dispersion of the data, only one direct relative was chosen per patient. Likewise, they were assured of anonymity in their responses. Family members were asked what their ethical position was in the event of needing to decide about appropriate therapeutic effort related to their relative.

When the patient participated, the interview was carried out in their patient cubicle, which in our unit is individual; thus, the confidentiality of their responses was maintained. None of the patients who participated had perceptual or cognitive impairment or neurosensory alterations and were not under the effects of medications that alter the ability to respond. The patients had to answer what their position was in a possible situation where appropriate therapeutic effort should be considered. Both patients and relatives answered the same questions. The average time spent per interview was about 45 min.

### 2.6. Statistical Analysis

A descriptive analysis of the data was carried out, calculating percentages and absolute frequencies for qualitative variables. For quantitative variables, the mean and standard deviation were calculated if they followed a normal distribution, and in the case of non-normality, the median and interquartile range were calculated. Inferential statistics were also performed using Pearson’s χ^2^ test. This test was used to assess an association or dependence between two categorical variables. Parametric test for 2/more than 2 independent samples for comparison of means after assessing homoscedasticity using Levene’s test. For the analysis between ordinal and qualitative variables, the Mann–Whitney U test was used. Finally, Spearman’s Rho coefficient was used for the bivariate analysis between quantitative variables. A *p* < 0.05 was considered statistically significant. All data were analyzed using the statistical software package SPSS 23.0 for Windows (SPSS Inc., Chicago, IL, USA).

### 2.7. Ethical Considerations

The present study was approved by the Hospital’s Clinical Research Ethics Committee (Reference C-128). All participants voluntarily provided written informed consent to accept their participation in this study. The study was carried out in accordance with good clinical practice criteria and the Declaration of Helsinki.

## 3. Results

**Figure 1:**
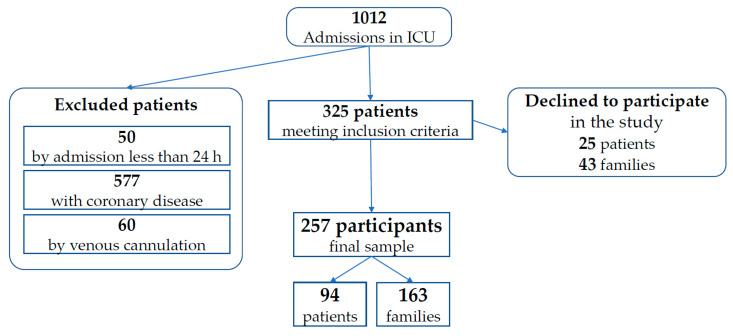
Flowchart of the participants’ selection process.

A total of 257 interviews were conducted: 94 directly to ICU patients able to respond by themselves and 163 answered by direct family members of patients admitted to the ICU (Figure 1).

### 3.1. Patients/Family Characteristics

Different features of the participants in the present study are shown in Table 1. One in four patients had no completed formal schooling compared to only 1% (*n* = 2) of relatives. Among the relatives, 60% (*n* = 98) had secondary or higher education compared to 34% (*n* = 32) of the patients (*p* = 0.001). Regarding religion, 54.5% (*n* = 140) of the interviewees were non-practicing Catholics compared to 29.2% (*n* = 75) practicing Catholics, and 14.4% had no religious affiliation (*n* = 37).

Most patients (83.6%, *n* = 215) lived at home independently, while 16.4% (*n* = 42) lived with relatives and were dependent. More women (16.9%, *n* = 21) were dependent compared to men (8.3%, *n* = 11), (*p* = 0.06). Around 26% (*n* = 13) of the dependent patients were admitted to the ICU for respiratory pathologies.

The majority (61%, *n* = 98) of the interviewed relatives were direct descendants, 36% (*n* = 58) daughters and 25% (*n* = 40) sons. The partner was present in 16% (*n* = 25) of the cases.

### 3.2. Clinical Profile

Three out of ten patients (28.7%) had already been admitted to the ICU before. Among the group of medically related admissions, 40% (*n* = 6) of the patients diagnosed with sepsis had previously been admitted to the ICU, followed by patients with cardiac and pulmonary conditions.

Most admissions were due to medical conditions (89.5%, *n* = 230), while surgical admissions represented the 10.55 (*n* = 27) of participants (Table 2). The incidence of cardiovascular disease was 25 points higher among the patients who responded to the questionnaire. The incidence of neurological pathology was five times higher in patients whose relatives responded to the survey (*p* = 0.011).

The median Karnofsky index (KI) was 90 (Interquartile Range (IQR) 75–100), with a minimum of 40 and a maximum of 100. The Barthel median was 100 (IQR 90–100), with a minimum of 40 and a maximum of 100. Around 14.3% (*n* = 37) had a KI lower than 60. KI scores below 60 indicate that the patient is unable to meet most of their basic needs. Results were similar for men and women, with 16.1% (*n* = 20) of the women not able to meet their basic needs compared to 12.7% (*n* = 17) of the men, according to the KI (*p* = 0.4). According to the Barthel index, 3.1% (*n* = 8) of the patients had moderate dependence, 32.7% (*n* = 84) mild dependence, and 64% (*n* = 165) were independent. Among women, 35.5% (*n* = 44) had mild dependence and 3.2% (*n* = 4) moderate dependence. Among men, 30.1% (*n* = 40) had mild dependence and 3% (*n* = 4) moderate dependence according to the Barthel index (*p* = 0.6).

Health-related quality of life was measured using the PAEEC scale, obtaining a median of 2 (IQR 0–5). Following the recommendations of the authors who validated the PAEEC scale, this variable was transformed into three categories: The score (0–2) corresponds to a good quality of life, a score (3–7) indicates a slight alteration in the quality of life, and a score greater than or equal to 8 indicates a moderate-severe deterioration in the quality of life prior to admission to the ICU.

According to this scale, 34% (*n* = 113) of the admitted patients experienced deterioration in their quality of life prior to ICU admission while 18.3% (*n* = 47) moderate-severe deterioration. When comparing gender, 26.3% (*n* = 35) of the men had a slight deterioration in their quality of life prior to admission compared to 25% (*n* = 31) of the women; however, 23.4% (*n* = 24) of the women had moderate-severe deterioration in their quality of life compared to 13.5% (*n* = 18) of men. The results of the quality of life survey according to the response of relatives or patients can be seen in Table 3.

An inverse correlation was found between the KI and the PAEEC quality of life scale in critically ill patients, Spearman’s Rho coefficient −0.8 (*p* = 0.001). Patients classified as unable to satisfy most of their needs according to the KI, had high scores on the PAEEC scale, that is, worse quality of life (*p* = 0.001). An inverse correlation was also found between the Barthel index and the PAEEC scale, Spearman’s Rho coefficient −0.7 (*p* = 0.001). According to the Barthel index, the patients classified as having some degree of dependency had high scores on the PAECC Scale, i.e., poorer quality of life (*p* = 0.001).

There were differences in the perception of health-related quality of life according to the type of respondent (*p* = 0.0001).

### 3.3. Responses to the Questionnaire

Around 54% (*n* = 88) of the family members knew the patient’s wishes on the limitation of treatment in the event of a poor prognosis. No differences were found between the type of familial relationship and knowledge of the patient’s opinion on treatment limitation (*p* = 0.6).

More than half (61.7%, *n* = 58) of the patients favored organ donation, while only 19% (*n* = 31) of the family members reported knowing this. Among direct relatives, 80.7% (*n* = 46) of daughters, 87.8% (*n* = 36) of sons, 45.5% (*n* = 5) of husbands, and 66.7% (*n* = 10) of wives were unaware of their relative’s opinion about organ donation. Only 9% (*n* = 5) of the patients in favor of the donation have formalized their wishes legally. Regarding the family members who knew the patient’s wishes regarding organ donation, only 22.6% (*n* = 7) knew if the patient had formalized this wish legally. Around 31% (*n* = 29) of the patients interviewed reported having a legal will. Among family members, 45.4% (*n* = 74) were aware of the presence of this official document (Table 4).

An interesting finding was to observe that both 100% (*n* = 11) of the husbands and 100% (*n* = 15) of the wives did not know whether their partner had signed an advanced healthcare directive.

No differences were found between the gender of the participants and the knowledge of their relative’s willingness to donate (*p* = 0.8) or the knowledge about the possession of an advanced healthcare directive by their relatives (*p* = 0.7).

Both patients and their families almost unanimously consider that they should be considered when making decisions about procedures and treatments.

More than half (62%, *n* = 102) of the interviewed family members believe that they should not fight for the patient’s life when irretrievable. In contrast, 54.2% (*n* = 51) of patients thought the same. Among patients, 51.9% (*n* = 14) of women and 43.3% (*n* = 29) of men believed that they should fight for life, even if it is irretrievable (*p* = 0.4).

Among direct relatives, 73% (*n* = 30) of the sons and 63.3% (*n* = 36) of the daughters believed they should not fight for the patient’s life when irretrievable. However, 63% (*n* = 7) of the husbands and 53.9% (*n* = 8) of the wives believe that the patient should fight for life, even if irretrievable.

The data corresponding to the decision to limit treatment in case of poor prognosis can be seen in Table 5. Among patients, 51.9% (*n* = 14) of the women would not accept the decision to limit compared to 38.8% (*n* = 26) of the men who would not accept this decision either (*p* = 0.2).

Of the patients classified with a moderate-severe alteration in the quality of life according to the PAEEC scale, 57.4% (*n* = 27) believed that they should not fight for the patient’s life when it is irretrievable, accepting treatment limitation. Similarly, 63.6% (*n* = 42) of patients with a mild deterioration in the quality of life thought the same. The data corresponding to the attitude of treatment limitation acceptance according to previous quality of life can be seen in Table 6.

**Figure 2:**
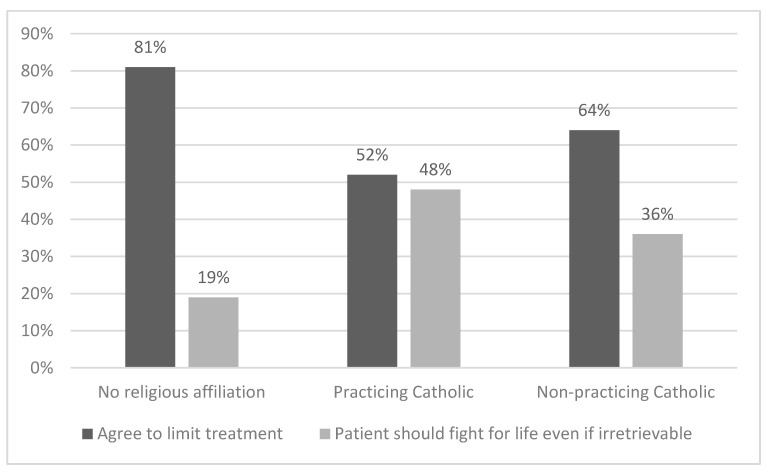
Attitude toward treatment limitation by religious affiliation.

In global terms, those not affiliated with any religion were approximately half as likely to fight for life, even if it is irrecoverable, than Catholics, and 1.5 times more likely to agree to limit treatment (*p* = 0.025 and 0.014, respectively) (Figure 2).

**Figure 3:**
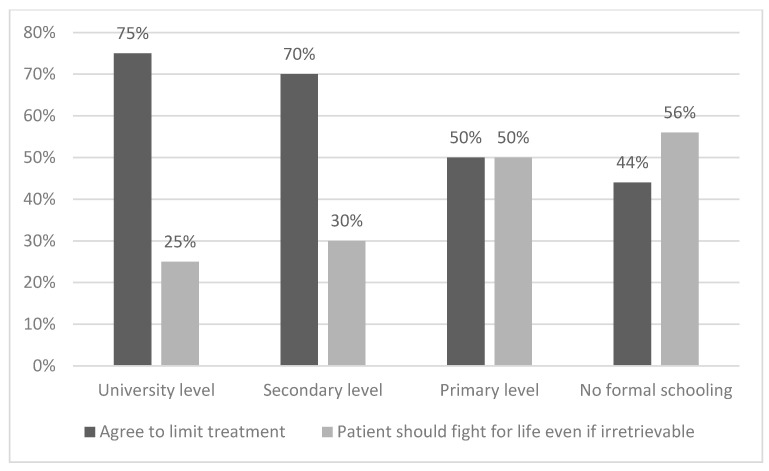
Attitude toward treatment limitation by education level.

Education level also highly influenced views on this subject: the higher the educational level, the lower the agreement with fighting for life when it is irretrievable, and the greater the agreement with limiting treatment in case of poor prognosis (*p* = 0.001 and 0.009, respectively) (Figure 3).

## 4. Discussion

### 4.1. Main Findings of the Study

The present study has analyzed the opinion and attitude of patients and their families regarding limitation of therapeutic effort and advanced directives in critical patients and whether end-of-life planning has been considered by patients. Our results show that ca. 60% of participants would agree to limit treatment in the case of a poor prognosis, independently of the degree of alteration in their quality of life. Less than 2% of participants had prepared advanced healthcare directives. Religion and educational level were determining factors for the choice of procedures at the end of life.

### 4.2. Context of the Findings within the Current Literature

LTE is a common practice for professionals working in ICUs worldwide. Among the elements to consider, it is important to evaluate the treatment possibilities and consider the participation of the patient or their representative in decision making [32,33,34]. However, there are differences between professionals, family members, and patients in the perception of LTE practice [35].

Patient’s quality of life is one of the main considerations when deciding whether to apply LTE. This concept can be considered to depend on the patient’s perspectives and can influence the patient’s limits. Therefore, its evaluation is carried out through surveys and validated indices, which are sometimes not feasible to use in critically ill patients or those presenting cognitive impairment [36,37].

In the present study, a considerable percentage of patients who had experienced deterioration in their quality of life before ICU admission was observed, with 18.2% of the participants experiencing moderate-severe deterioration. Moreover, 14.3% had a KI of less than 60, indicating the inability to satisfy most of their basic needs. This is in line with several published studies, where one of the fundamental factors that guided patient decision making was their previous quality of life and future or predictable quality of life [38]. The future quality of life is presented in research as an important factor to be considered for LTE. The way patients or relatives evaluate the quality of life often differs, as is clear in previous literature [39] and in our research, in which the perception of quality of life is significantly worst for family members than for the patient itself. Therefore, to minimize this difference, patients and their families must have adequate information about their condition and prognosis to decide what risks they would be willing to take, as previously suggested [40].

Our study, in agreement with others [41], reveals that advanced healthcare directives (AHD) are not usually done by critical patients, although there are countries, e.g., English-speaking, where the establishment of AHD has a greater presence in the population [42]. However, relatives may have some kind of information about the preferences of the patient concerning the end of life. Perhaps, since 28.7% of the patients have had previous admissions to the ICU, this previous experience could have influenced a change in attitude about limiting therapeutic processes in terminal situations. These data are consistent with those observed in other studies where patients, even without having formalized AHD, state that they have discussed and commented on aspects related to end-of-life care with their closest relatives [43]. Other authors point out that AHD were little used, and only three out of ten of the family members knew about them [44]. AHDs rates vary between countries; in the US rates are higher than those found in Europe, as shown by Kumar et al. [45], where having AHD reaches 22%. Therefore, our results indicate that patients do not prepare for the dying process sufficiently in advance, either in the most usual way of registering the legal will or by making an AHD document or by appointing a legal representative, which is in line with previous reports [46].

Six out of ten participants in our cohort would agree to limit treatment in case of poor prognosis, especially those who had a previous deterioration in their quality of life. These results are in line with Ortiz Goncalves [47], where more than 50% of the studied population were against therapeutic obstinacy. As shown in other research, the reason for this attitude could be related to the patient’s interest in avoiding pain and suffering, the desire not to be a burden for family members, and losing their independence [38]. An essential consideration is to determine whether the families’ opinion coincides with the wishes of the patients. Studies have shown that the opinion of the families may differ from the opinion of the patient [39], and they may not know what decision they would make [48]. In the present work, this difference is especially evident and significant concerning the willingness to be a donor with a positive response of 61.7% of patients versus 19% of family members. However, we do not detect significant differences in the acceptance of treatment limitation between both groups, although only 54% (*n* = 88) of the family members knew the patient’s wishes on limitation of treatment in the event of a poor prognosis. In the systematic review by Shalowitz et al. [49], the degree of concordance between the patient’s decision and that of their relatives was only 68%, making research in this field relevant.

It should be noted that 37.7% of those interviewed had a proactive attitude, i.e., they consider that patients should fight for life, even if irrecoverable, and favored maintaining treatments. This finding agrees with those of other authors who highlight the interventionist attitude on the part of family members even in elderly patients or those with cognitive impairment [50,51].

The results obtained also show that the participants had favorable attitudes toward organ donation, although four out of ten patients were against donation, consistent with other research [52]. These results could be related to the education level of the patients, as it is known that people with a higher educational level donate the most compared to those with a lower educational level [53]. Regarding patient families, the majority did not know the wishes of their relatives related to organ donation, as stated above, probably because of fear of talking about death and related aspects or due to unknowing the process of organ donation [54].

Our study shows that the higher the educational level, the lower the desire to fight for life when it is irretrievable and the greater the agreement to limit treatment. Having a high level of education is significantly associated with a better understanding of clinical information and a greater predisposition to make decisions contrary to therapeutic obstinacy [55,56].

In our work, those participants without any religious affiliation were much less likely to consider fighting for life, even when irretrievable, than Catholics, and more likely to agree to limit treatment. Religion has become one of the determining factors in the choice of procedures at the end of life, both for patients and their relatives, and is a variable that significantly correlates with end-of-life preferences [57,58,59].

### 4.3. Limitations of the Study

The present study is a unicentric study with the limitation that this implies for generalizing the conclusions beyond the context of the study itself. In addition, the impossibility of carrying out the questionnaire in all patient cases, including patients’ family members instead, was an unavoidable limitation in some cases (21%), probably due to the critical status of patients when they enter the ICU. Conversely, to minimize any impact of interviewer bias, data were collected through an interview by the same professional, which facilitated the homogeneity of the explanation of the concepts and decreased variability in information collection. Another limitation was the impossibility of comparing whether the wishes of the patients coincided with the perspectives of their own families.

### 4.4. Conclusions

Six out of ten participants in our cohort would agree to limit treatment in the case of poor prognosis, many considering their relative’s wishes, especially those who had previous deterioration in the quality of life. Our results also indicate that patients do not prepare for the process of dying well in advance, either in the most usual way of registering a legal will or by making an advanced directive document. Religion and educational level become determining factors in the choice of procedures at the end of life, both for patients and their families. A practical/clinical implication of our study is that it should consider the patient’s point of view in this situation and take into account not only the decision of the healthcare staff but also of the family to provide high-quality end-of-life care [60].

## Figures and Tables

**Table 1:** Patients/Family Characteristics.

		Total		Family		Patient		
		N	%	N	%	N	%	*p*-Value χ^2^
**GENDER**	**Men**	133	51.8	66	40.5	67	71.3	0.001
	**Women**	124	48.2	97	59.5	27	28.7	
**AGE (years)**				45.6 + 11.7		59.6 + 13.4		
**EDUCATION LEVEL**	**No education**	25	9.7	2	1.2	23	24.5	0.001
	**Primary level**	102	39.7	63	38.7	39	41.5	
	**Secondary level**	78	30.4	57	35.0	21	22.3	
	**University level**	52	20.2	41	25.2	11	11.7	
**RELIGION**	**Non-practicing Catholic**	140	54.5	87	53.4	53	56.4	0.457
	**Practicing Catholic**	75	29.2	50	30.7	25	26.6	
	**No religion**	37	14.4	24	14.7	13	13.8	
	**Orthodox**	2	0.8	1	0.6	1	1.1	
	**Evangelist**	2	0.8	0	0.0	2	2.1	
	**Muslim**	1	0.4	1	0.6	0	0.0	

**Table 2:** Admission Diagnosis in ICU.

		*n*	%
MEDICAL PATHOLOGY	**Cardiovascular**	98	38.1
	**Respiratory**	63	24.5
	**Neurological**	28	10.9
	**Sepsis**	15	5.8
	**Gastrointestinal**	11	4.3
	**Renal**	5	1.9
	**Traffic accident**	4	1.6
	**Traumatology**	2	0.8
	**Neoplasia**	1	0.4
	**Heatstroke**	1	0.4
	**Self-harm**	1	0.4
	**Metabolic acidosis**	1	0.4
SURGICAL PATHOLOGY	**Gastrointestinal surgery**	8	3.1
	**Traumatology**	6	2.3
	**Neurosurgery**	5	1.9
	**Urology**	3	1.2
	**Vascular surgery**	2	0.8
	**Gynecological surgery**	2	0.8
	**Otorhinolaryngology**	1	0.4
	**TOTAL**	**257**	**100**

**Table 3:** Distribution of health-related quality of life according to questionnaire respondents.

RESPONDENTS			PAEEC SCALE SCORE			Total
			0–2 Points	3–7 Points	>8 Points	
	**Family**		75 (46%)	51 (31.3%)	37 (22.7%)	163 (100%)
	**Patient**		69 (73.4%)	15 (16%)	10 (10.6%)	94 (100%)

The score (0–2 points) corresponds to a good quality of life, a score (3–7 points) indicates a slight alteration in the quality of life, and a score greater than or equal to 8 points indicates a moderate-severe deterioration in the quality of life.

**Table 4:** Patients’ and relatives’ responses regarding organ donation, advance directives, healthcare proxy, and the possession of a legal will.

								*p* Value χ^2^
		Total		Family		Patient		
		N	%	N	%	N	%	
**Willingness to be a donor**	**No**	168/257	65.4	132/163	81.0	36/94	38.3	<0.0001
	**Yes**	89/257	34.6	31/163	19.0	58/94	61.7	
**Formalized donor status**	**No**	75/87	86.2	24/31	77.4	51/56	91.1	0.077
	**Yes**	12/87	13.8	7/31	22.6	5/56	8.9	
**Healthcare proxy**	**No**	233/256	91.0	148/162	91.4	85/94	90.4	0.801
	**Yes**	23/256	9.0	14/162	8.6%	9/94	9.6	
**Advanced Healthcare Directive**	**No**	253/257	98.4	160/163	98.2	93/94	98.9	0.628
	**Yes**	4/257	1.6	3/163	1.8	1/94	1.1	
**Legal Will**	**No**	154/257	59.9	89/163	54.6	65/94	69.1	0.022
	**Yes**	103/257	40.1	74/163	45.4	29/94	30.9	

**Table 5:** Acceptance of treatment limitation in the event of a poor prognosis or poor future quality of life.

QUESTIONNAIRE RESPONDENTS	WOULD NOT ACCEPT	WOULD ACCEPT	*p*-Value χ^2^
**Family**	57/163 (34.9%)	106/163 (65.0%)	0.227
**Patient**	40/94 (42.5%)	54/94 (57.4%)	
**Total**	97/257 (37.7%)	160/257 (62.2%)	

**Table 6:** Attitude toward treatment limitation according to quality of life prior to ICU admission.

PAEEC SCALE SCORE * (Points)	DECISION TO LIMIT TREATMENT		*p*-Value U Mann–Whitney
	Would Not Accept	Would Accept	
0–2	58 (40.3%)	86 (59.7%)	0.5001
3–7	21 (31.8%)	45 (68.2%)	
≥8	18 (38.3%)	29 (61.7%)	
TOTAL	97 (37.7%)	160 (62.3%)	

* The score (0–2) corresponds to a good quality of life, a score (3–7) indicates a slight alteration in the quality of life, and a score greater than or equal to 8 indicates a moderate-severe deterioration in the quality of life.

## Data Availability

Data are available in the corresponding tables and figures.
